# LATE EVALUATION OF PATIENTS UNDERGOING MANIPULATION OF THE KNEE AFTER TOTAL ARTHROPLASTY

**DOI:** 10.1590/1413-785220172506165770

**Published:** 2017

**Authors:** PEDRO GUILME TEIXEIRA DE SOUSA, YURI LUBIANA CHISTÉ, RODRIGO SATTAMINI PIRES E ALBUQUERQUE, HUGO ALEXANDRE DE ARAÚJO BARROS COBRA, JOÃO MAURÍCIO BARRETTO, NAASSON TRINDADE CAVANELLAS

**Affiliations:** 1. Knee Surgery Center at the Instituto Nacional de Traumatologia e Ortopedia, Rio de Janeiro, RJ, Brazil.

**Keywords:** Arthroplasty, replacement, knee/methods. Manipulation, orthopedic. Knee joint. Range of motion, articular., Artroplastia do joelho/métodos, Manipulação ortopédica, Articulação do joelho, Amplitude de movimento articular.

## Abstract

**Objective:**

We compared gains in range of motion in patients who underwent manipulation within 12 weeks of total knee arthroplasty (TKA) and after this period. We also evaluated maintenance of the arc obtained from knee manipulation in late follow-up, along with factors associated with poorer outcomes.

**Method:**

The study was divided into two groups according to the time after TKA; the surgeries took place between January 2008 and December 2014.

**Results:**

When comparing the range of motion between early and late manipulations, the group that underwent manipulation within 12 weeks of the TKA exhibited better outcomes, but these were not statistically significant. We observed that 14.3% of cases retained the same range attained at the time of manipulation. In late evaluation after manipulation, 47.7% of the sample had a range of less than 90 degrees. The significant risk factors for recurrence of knee stiffness in the long term are poor range of motion before TKA and before manipulation, female sex, and secondary arthritis.

**Conclusion:**

Women previously diagnosed with secondary osteoarthritis and poor range of motion before TKA or manipulation are at higher risk for late stiffness. **Level of Evidence III, Retrospective Comparative Study.**

## RESUMO

**Objetivo:**

Comparar o ganho de arco de movimento entre os pacientes submetidos à manipulação antes de 12 semanas pós-artroplastia total do joelho (ATJ), e depois desse período. Além disso, avaliar tardiamente a manutenção do arco obtido com a manipulação do joelho e fatores relacionados com os piores resultados.

**Método:**

O estudo foi dividido em dois grupos, de acordo com o tempo pós-ATJ. Os procedimentos ocorreram entre janeiro de 2008 e dezembro de 2014.

**Resultados:**

Quando comparamos os arcos de movimento entre as manipulações precoces e tardias, o grupo submetido à manipulação em 12 semanas da ATJ apresentou melhores resultados, porém, sem significância estatística. Foi observado que 14,3% dos casos mantiveram a mesma amplitude alcançada no momento da manipulação. Na avaliação tardia, 47,7% da amostra obtiveram amplitude menor que 90 graus. Os fatores de risco significantes para recidiva tardia de rigidez são arco de movimento ruim antes da ATJ e antes da manipulação, sexo feminino e artrites secundárias.

**Conclusão:**

Mulheres com diagnóstico prévio de osteoartrite secundária e com arco ruim antes da ATJ ou da manipulação têm maior risco de rigidez tardia. **Nível de Evidência III, Estudo Retrospectivo Comparativo.**

## INTRODUCTION

Total knee arthroplasty (TKA) is a highly effective surgical procedure for treating knee arthrosis; it significantly improves patient quality of life by relieving symptoms and restoring joint function.^1^ ^,^ ^2^ Despite good results and constant advances in implant characteristics, surgical techniques, and postoperative recovery protocols, some patients have poor functional outcomes, which restrict their activities of daily living.^3^ ^,^ ^4^

More than 20% of patients who undergo TKA may develop stiffness, and consequently an arc of motion with less than 90° flexion.^5,6^ A variety of factors have been described as influencing the occurrence of this complication; these include having a poor range prior to surgery, low socioeconomic levels, diabetes mellitus, lack of patient compliance to the post-surgical rehabilitation, and previous arthroplasty of the knee.^5^ ^,^ ^7^

There is no consensus in the literature precisely defining the arc of functional movement. In general, 90° flexion has been considered a minimal functional recovery after TKA. Not obtaining this mobility can be devastating, and negatively affects activities of daily living and patient satisfaction. Biomechanical studies have demonstrated the minimal ranges of flexion to perform some activities, such as 83° to go up and down stairs, 93° to sit, and 65°-70° to perform the swing phase of the gait cycle.^2^ ^,^ ^5^ ^,^ ^8^

Among the various options to treat stiffness after TKA, manipulation under anesthesia has been considered the first line of treatment after other non-surgical measures such as physiotherapy fail.^2^ ^,^ ^5^ ^,^ ^8^ Nevertheless, the long-term results of this method have not been well studied. There is also no consensus in the literature regarding the ideal time to perform manipulation.^5^ ^,^ ^9^ Some authors have suggested that between 2 and 12 weeks post-surgery is the ideal time to perform manipulation, since a more invasive procedure would be required after this time due to maturation of the scar tissue.^5^ ^,^ ^9^ ^-^ ^11^ Meanwhile, other studies found no differences in gains of range of motion between a group that received early treatment (within twelve weeks of surgery) and a late-treatment group (manipulation more than twelve weeks after surgery).^5^ ^,^ ^8^

The objective of this study is to compare gains in range of motion between patients who received early manipulation (within 12 weeks of TKA), and those who received manipulation after this period. In addition, we assessed maintenance of the arc obtained from knee manipulation over the medium and long terms, and factors related to poorer outcomes.

## MATERIALS AND METHODS

This is a historical cohort or retrospective study, in which patients who underwent knee manipulation under anesthesia to treat joint stiffness after total knee arthroplasty were selected according to the following inclusion criteria: TKA performed at our institute; TKA performed between January 2008 and December 2014; at least one year between knee manipulation and reassessment; procedures performed in accordance with the routine of the hospital’s knee surgery group, as described below. The knee was x-rayed before indicating manipulation, in order to assess the size and positioning of the implant.

According to the knee surgery group routine, patients with primary TKA were approached in the initial intervention via medial parapatellar access, using ischemia via a pneumatic cuff. The pneumatic cuff was placed on the leg and inflated to 100 mmHg above systolic pressure minutes before the skin incision. This same pressure was maintained for up to 2 hours, on average, and the tourniquet was then deflated. We reviewed hemostasis, closed the wound by planes, and placed an extrarticular drain in a closed suction system. All patients were subjected to the same prophylaxis protocol for infection and deep vein thrombosis. All received guidelines and a schematic post-surgical rehabilitation protocol, in addition to monitoring with physical therapy at home or in the institute’s rehabilitation department.

The manipulations were performed under sedation and a peripheral femoral block. The patient was positioned on the surgical table in dorsal decubitus with the muscles relaxed as much as possible. The hip was positioned in 90^0^ of flexion and the tibia was stabilized in the proximal region, and the knee was flexed slowly and gently.^6^

Both procedures were performed by orthopedists from the knee surgery center at the National Institute of Traumatology and Orthopedics (INTO). After the manipulation, a control X-ray was taken for medical documentation. The range of motion prior to manipulation and after the procedure was confirmed by the surgeon in charge and documented in surgical record.

All cases that met any of the following criteria were excluded: patients in whom manipulations were performed after other surgical procedures (non-TKA) performed at INTO; manipulations that developed immediate complications, such as periprosthetic fractures or deep vein thrombosis, which hindered rehabilitation and maintenance of the range of motion obtained during the manipulation; patients with less than one year of follow-up; patients with incomplete medical documentation.

The included patients returned for a follow-up appointment in which the maintenance of the range of flexion obtained from the manipulation was assessed, along with the Knee Society Score (KSS).^12^ Demographic and clinical data were collected from the pre-, intra-, and postoperative periods via interviews and the medical records.

The patients who returned for follow-up were divided in groups according to the time elapsed between arthroplasty and manipulation: Group 1: patients who underwent early manipulation, within 12 weeks of TKA. Group 2: patients who underwent late manipulation, more than 12 weeks after TKA.

The implants used in the TKA varied between patients, and included PFC Sigma, TC3, and Natural Knee implants; the platform, type of stabilization, cementing, and placement of the patellar component also varied.

The study was approved in advance by the institutional review board (CAAE: 52871916.6.0000.5273). Participants were invited to participate in the study and asked to sign the informed consent form. From the collected data, we constructed a bank of data we analyzed using SPSS (Statistical Package for the Social Sciences) version 22.0 and Microsoft Excel 2007 software.

Fisher’s exact test and the nonparametric Mann-Whitney test were used to compare the early and late manipulations groups for qualitative and quantitative variables, respectively. The p-values (all greater than 5%) did not exhibit significant differences in the qualitative variables (patient and surgery characteristics).

## RESULTS

During the study period, 2865 knee total arthroplasties were performed, and a total of 45 patients underwent manipulation of the knee under anesthesia after total arthroplasty. After analysis of the inclusion and exclusion criteria, 6 patients were excluded: 2 had incomplete medical documentation, and 4 developed complications after the knee manipulation procedure. Of the 39 remaining patients, 3 underwent bilateral manipulation, totaling 42 manipulations; 16 of these procedures (38.1%) were performed in men, and 26 (61.9%) in women. The mean patient age was 62.2 years, ranging from 45 to 83 years. The majority of patients were classified as ASA II (78.6%), and hypertension was the most frequent comorbidity (66.7%).

The most common indication for TKA was primary osteoarthritis (71.4% of the cases), followed by rheumatoid arthritis (16.7%), sequelae of fracture (7.1%), hemophilic arthritis (2.4%), and sequelae of tuberculosis (2.4%). The most commonly used brand of implant was the PFC Sigma (88.1%). Only two individuals received arthroplasty with a semi-constrained implant, the TC3. Manipulation under sedation was most frequent between 7 and 12 weeks after TKA (59.5% of cases). Manipulation was performed within six weeks of the TKA in 26.2% of cases, and only in 6 cases (14.3% of the sample) was the manipulation performed late, between 13 and 26 weeks after TKA.

**Figure 1:**
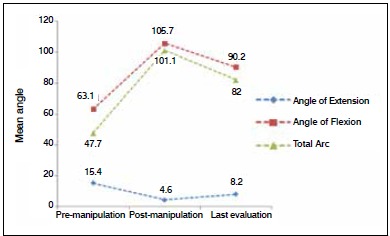
Change in mean angle of arc of motion at three distinct times.

The arc of motion (maximum length, maximum flexion, and sum of arc) was measured at three different times: before manipulation, after manipulation, and in the ambulatory follow-up assessment. Figure 1 shows the change in the mean values for flexion and extension angles, as well as the total arc of motion at each assessment.

**Figure 2:**
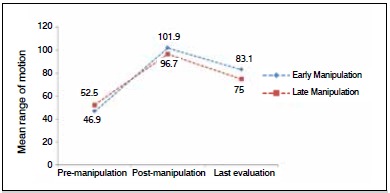
Change in mean arc of motion for the early and late manipulation groups.

**Figure 3:**
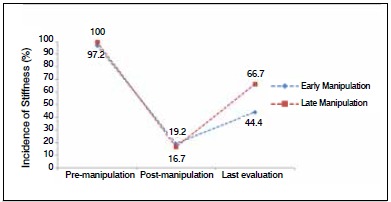
Incidence of stiffness in the study groups (Arc < 90°).

When we compared the ranges of motion from early manipulation with late manipulations, we found better results in the values for cases when manipulation was performed before 12 weeks. However, these values were not statistically significant, which can be explained by the small sample size of the group in which late manipulation was performed. (Figure 2) When we considered the incidence of knee stiffness (arc < 90°) in the long term, we found considerably higher recurrence in the late-treatment group, as shown in Figure 3.

The follow-up time between the completion of the knee manipulation and the outpatient evaluation to collect the data ranged from 12 to 81 months. Table 1 shows the frequency of cases that maintained the arc achieved from manipulation to the time of the outpatient assessment. Only 14.3% of the cases maintained the same range which was achieved in manipulation. Considering a variation of 10% in the arc from manipulation, 33.3% maintained this range of motion at reassessment. If this is adjusted to a margin of 10 degrees of difference, the incidence increased to 35.7%. Only one patient in the late manipulation group maintained the same arc after manipulation. Despite the differences between the groups, Fisher’s exact test did not detect a statistically significant difference.

**Table 1:** Frequency of cases that maintained the arc obtained after manipulation in the long term.

Group	Maintained the same arc after manipulation	Maintained the same arc with variation of up to 10%	Maintained the same arc with up to 10 degrees of difference
Total (%)	6 (14.3%)	14 (33.3%)	15 (35.7%)
Early Manipulation (%)	5 (13.9%)	13 (36.1%)	14 (38.9%)
Late Manipulation (%)	1 (16.7%)	1 (16.7%)	1 (16.7%)
P-value from Fisher's Exact Test	1.000	0.645	0.395

At the outpatient evaluation, the Knee Society Score (KSS)^12^ values were calculated for the early and late manipulation groups. This evaluation combines subjective and objective information and separates the knee score (pain, stability, range of motion, among other components) from the patient’s functional score (ability to walk and go up and down stairs). A significant difference was found between the knee scores for the groups in question. The p-value was 0.027, showing that the knee scores for the late manipulation group were significantly lower than those of the early manipulation group. (Table 2)

**Table 2:** Comparison between groups via KSS.

Variable	Manipulation	Mean	Median	Standard Deviation	P-value from Mann-Whitney Test
KSS for knee	Early	81.2	88	14.4	0.027
	Late	70.7	69	6.7	
KSS patient functional score	Early	72.4	78	18.2	0.103
	Late	61.7	60	12.5	

In the outpatient follow-up assessment, we perceived that stiffness (amplitude of less than 90 degrees) was present in 20 cases (47.7% of the sample). In addition, we compared the variables collected between patients with and without stiffness, in an attempt to find some statistically significant risk factor for limited range of motion in the long term, even after manipulation under anesthesia.

When comparing the ranges of motion before TKA and prior to manipulation, we noted that the values in patients who developed stiffness were significantly lower. (Table 3) Other significant risk factors were sex and indication for arthroplasty; women are 8.2 times more likely than men to develop stiffness after manipulation. As for the indication for TKA, the percentage of patients who undergo this procedure to treat primary osteoarthritis and develop stiffness in the long term (36.7%) is significantly lower than the percentage of patients who undergo TKA for another reason (75.0%). The odds ratio is 0.2, with a 95% confidence interval. Table 4 also shows other qualitative variables that had no statistical significance.

**Table 3:** Comparison of quantitative variables in patients with and without stiffness after manipulation.

Median of Variable	Post-manipulation stiffness		
	No	Yes	P-value from Mann-Whitney Test
Pre-TKA arc	95º	75º	0.010
Pre-manipulation arc	55º	32.5º	0.020

**Table 4:** Association between qualitative variables and stiffness after manipulation.

Variable	Qualification (subgroup) of the Variable	Number of cases in the Stiffness after Manipulation subgroup	Percent of cases in the Stiffness after Manipulation subgroup	P-value from Chi-squared Test comparing the frequency of the subgroups
Sex	F	17	65.4	0.003
	M	3	18.8	
ASA (CCECK)	1	3	33.3	0.460*
	2	17	51.5	
DM	No	16	45.7	0.691*
	Yes	4	57.1	
HBP	No	7	50.0	0.827
	Yes	13	46.4	
RA	No	15	42.9	0.229*
	Yes	5	71.4	
Smoking	No	18	47.4	1.00*
	Yes	2	50.0	
Has Any Comorbidity	No	2	28.6	0.414*
	Yes	18	51.4	
Indication for TKA: Primary Arthritis	No	9	75.0	0.040
	Yes	11	36.7	
Indication for TKA: Sequel of fracture	No	18	46.2	0.598*
	Yes	2	66.7	
Indication for TKA: Rheumatoid Arthritis	No	15	42.9	0.229*
	Yes	5	71.4	
Implant platform	Rotating	13	41.9	0.298*
	Fixed	7	63.6	
Patella substituted	No	8	40.0	0.374
	Yes	12	54.5	
Time at which manipulation under sedation was performed (Weeks)	0 to 6	6	54.5	0.435**
	7 to 12	10	40.0	
	13 to 26	4	66.7	
Late Manipulation	No	16	44.4	0.400*
	Yes	4	66.7	

* Fisher’s exact test. ** Test inconclusive, we recommend increasing the samples in subgroups.

## DISCUSSION

Stiffness in the knee after TKA is a well-known problem that can lead to poor patient outcomes and limit activities of daily living in patients.^13^ The literature on this subject is somewhat controversial, starting with the definition itself. Fox and Poss^6^ defined stiffness as less than 90^0^ active knee flexion two weeks after TKA surgery. Other researchers such as Kim et al.^14^ defined rigidity as a capsular contracture greater than or equal to 15° or a flexion less than 75°.^14^ ^,^ ^15^ As a result, the literature is confusing and there are no studies with a high evidence.

The lack of a consensus on treatment or a standardized algorithm leads to other problems in the literature. Many forms of treatment have been described, including physiotherapy, knee manipulation under anesthesia, manipulation associated with arthroscopy, arthrotomy, and revision arthroplasty.^9^ Movement gains through physiotherapy are often modest, with studies showing an average gain of 5° in knees with arthrofibrosis after TKA.^9^ Manipulation under anesthesia is generally considered the initial surgical step in treating stiffness after TKA.^5^ When associated with arthroscopy, this procedure allows the surgeon to examine the implants and assess the presence of impact on soft tissue, loose bodies, or adhesions.^9^ ^,^ ^16^ Open release of adhesions or surgical revision are often used in refractory cases or in cases with poor positioning of the components.^9^

Our study only assessed patients subjected to manipulation under sedation in association with a femoral nerve block. Other studies have opted for general anesthesia;^6^ ^,^ ^8^ ^,^ ^9^ ^,^ ^15^ there is no evidence in the literature that the type of anesthesia used influences the final outcome of the manipulation. Choi et al.^2^ defended regional anesthesia as an improvement factor for the results of manipulation after TKA.

There is no consensus in the literature about the most appropriate time to perform surgical manipulation after TKA. Consequently, our research is pertinent and relevant. A series of studies have shown superior results when manipulation is performed early.^3^ ^,^ ^5^ ^,^ ^6^ ^,^ ^9^ Many authors consider 12 weeks post-TKA to be the deadline for manipulation, since a more invasive procedure is necessary after this time because of maturation of the scar tissue.^5^ ^,^ ^9^ ^-^ ^11^ However, some studies found no significant differences in gains in range of motion between early and late groups (undergoing manipulation before and after twelve weeks).^5^ ^,^ ^8^

When we compared the early and late groups in our study, a significant difference was seen between the mean KSS knee scores during the reevaluation in the medium and long term. This shows that although some patients did lose range over time, the functional score was still significantly higher in the group that underwent manipulation earlier. Issa et al.^5^ demonstrated a significant difference in KSS scores when comparing early and late groups before and after manipulation, but did not perform a long-term assessment. Since this present study was retrospective, it was not possible to compare scores before and after manipulation.

The mean patient age was 62.2 years, which is considered low for patients who undergo TKA. According to the study by Fox and Poss,^6^ more advanced age seems to be a factor in difficulty attaining range of motion after manipulation.

Although we did not find a statistically significant relationship between the implants used, the literature shows that they can directly affect the final results of the arc of motion.^6^ Studies show that prostheses which sacrifice the posterior cruciate ligament (PCL) demonstrate greater gain after manipulation than those which preserve this ligament.^2^ ^,^ ^15^ In our sample, the majority of cases involved TKA with sacrifice of the PCL (92.9%). The only three patients who received implants where the PCL was retained were handled early after the TKA, and made good progress after manipulation, all showing at least 100° of range of motion in the reassessment.

The time elapsed between the manipulation and patient reassessment ranged from 12 to 81 months, which according to the interpretation of Esler et al.^17^ can be considered a considerable clinical follow-up, since a minimal gain was observed after a period of 1 year.^2^ We correlate this good result with a minimum range of 90^0^, for the knee, as well as the research by Choi et al.,^2^ which was based on the idea that this is considered the minimum arc to perform basic activities.

When we look at the variables for patients who had knee stiffness (arc < 90°) in the outpatient assessment, we found some statistically significant variables for this outcome, such as the arcs of movement pre-TKA and pre-manipulation, female sex, and the indication for arthroplasty. As for the range of motion in the prior to the primary TKA surgery being a determining factor in the postoperative results, we found studies that agree^6^ ^,^ ^18^ and disagree ^2,8^ with this hypothesis. Several studies have shown a strong correlation between female sex and knee stiffness after manipulation,^2^ ^,^ ^5^ ^,^ ^6^ ^,^ ^9^ ^,^ ^15^ even though not all of these were statistically proven.

With regard to pre-TKA etiology, we found that patients undergoing this procedure for primary osteoarthritis have significantly less risk of stiffness in the long term. Consequently, the group formed by other indications (rheumatoid arthritis, sequel of fracture, hemophilic arthritis, and sequela of infection) was considered a risk factor. Some studies have shown a direct relationship between arthroplasties performed for secondary arthritis and stiffness after manipulation.^2^ ^,^ ^6^

Unfortunately, not all patients in our service were able to access the continuous passive movement device (CPM) because of cost. This tool directly impacts the maintenance of the range of motion achieved after manipulation.^2^ Physiotherapy is an essential complementary phase after orthopedic procedures. All the patients in our study received guidance via booklets given to them by our team physiotherapists, and the institute’s rehabilitation service was also available for post-procedure follow-up. Yoo et al.^3^ emphasized the need for aggressive physical therapy after manipulation to achieve good outcomes.

Our main limitation was the fact that this is a retrospective study. Since we did not find other studies with this line of research in the country, we believe that the issue requires further study, especially research with level I evidence.

## CONCLUSION

Knee manipulation under sedation is a procedure that can improve the functional outcomes of patients with knee stiffness after TKA, and presents better results in patients who undergo this procedure early. In the long-term follow-up, 14.3% of the patients maintained the range of motion they achieved from manipulation, and 47.7% of the sample developed a range of motion in the knee of less than 90 degrees. Patients at high risk for developing rigidity are women who underwent TKA to treat secondary osteoarthritis and already had poor range of motion before arthroplasty or before manipulation under anesthesia.
